# Characterization of *Helianthus annuus* Lipoic Acid Biosynthesis: The Mitochondrial Octanoyltransferase and Lipoyl Synthase Enzyme System

**DOI:** 10.3389/fpls.2021.781917

**Published:** 2021-11-18

**Authors:** Raquel Martins-Noguerol, Sébastien Acket, M. Adrián Troncoso-Ponce, Rafael Garcés, Brigitte Thomasset, Mónica Venegas-Calerón, Joaquín J. Salas, Enrique Martínez-Force, Antonio J. Moreno-Pérez

**Affiliations:** ^1^Departamento de Biología Vegetal y Ecología, Facultad de Biología, Universidad de Sevilla, Seville, Spain; ^2^UPJV, UMR CNRS 7025, Enzyme and Cell Engineering, Centre de Recherche Royallieu, Université de Technologie de Compiègne, Compiègne, France; ^3^Instituto de la Grasa-CSIC, Seville, Spain

**Keywords:** sunflower, octanoyltransferase, lipoyl synthase, lipoic acid, mitochondrial lipoylation

## Abstract

Lipoic acid (LA, 6,8-dithiooctanoic acid) is a sulfur containing coenzyme essential for the activity of several key enzymes involved in oxidative and single carbon metabolism in most bacteria and eukaryotes. LA is synthetized by the concerted activity of the octanoyltransferase (LIP2, EC 2.3.1.181) and lipoyl synthase (LIP1, EC 2.8.1.8) enzymes. In plants, pyruvate dehydrogenase (PDH), 2-oxoglutarate dehydrogenase or glycine decarboxylase are essential complexes that need to be lipoylated. These lipoylated enzymes and complexes are located in the mitochondria, while PDH is also present in plastids where it provides acetyl-CoA for *de novo* fatty acid biosynthesis. As such, lipoylation of PDH could regulate fatty acid synthesis in both these organelles. In the present work, the sunflower *LIP1* and *LIP2* genes (*HaLIP1m* and *HaLIP2m)* were isolated sequenced, cloned, and characterized, evaluating their putative mitochondrial location. The expression of these genes was studied in different tissues and protein docking was modeled. The genes were also expressed in *Escherichia coli* and *Arabidopsis thaliana*, where their impact on fatty acid and glycerolipid composition was assessed. Lipidomic studies in Arabidopsis revealed lipid remodeling in lines overexpressing these enzymes and the involvement of both sunflower proteins in the phenotypes observed is discussed in the light of the results obtained.

## Introduction

Lipoic acid (LA; 6,8-dithiooctanoic acid) is a sulfur containing coenzyme found in most bacteria and eukaryotic organisms. This co-factor is essential for the activity of several key enzymes involved in oxidative and single carbon metabolism, including pyruvate dehydrogenase (PDH), 2-oxoglutarate dehydrogenase (2-OGDH), branched-chain 2-oxoacid dehydrogenase (BCDH), acetoin dehydrogenase and the glycine cleavage system (Mooney et al., 2002). The activity of all these complexes requires the covalent binding of LA molecules to either the E2 subunit in the case of PDH, 2-OGDH, or BCDH, or to protein H of the glycine cleavage system.

Lipoic acid is generated from the octanoyl-acyl carrier protein (ACP) during *de novo* fatty acids biosynthesis through the activity of β-ketoacyl-(ACP) synthase (KAS). Octanoyl-ACP is the substrate of a two-step reaction catalyzed by the enzymes octanoyltransferase (LIP2) and lipoyl synthase (LIP1). In the first step, LIP2 transfers the octanoyl moiety from octanoyl-ACP to the corresponding *apo*-proteins, to which it binds through an amide bond. LIP2 acts as an acylated intermediate in this reaction, with the octanoate transferred from the ACP to a Cys residue of this enzyme and finally, to the target apo-protein (Zhao et al., 2005; Ma et al., 2006). In the second reaction, LIP1 inserts two sulfur atoms into the octanoyl chain, producing LA and hence, the lipoylated *holo*-subunit. LIP1 contains two distinct [4Fe–4S] clusters. The RS cluster, characteristic of the superfamily of enzymes that reduce *S*-adenosylmethionine (SAM), which is involved in SAM reduction, and that generates 5′-deoxyadenosyl radicals which attack the C-H bonds at position C6 and C8 of the octanoyl chain for further sulfur insertion. The second cluster is specific to lipoyl synthases and it is called an auxiliary cluster, which is thought to act as the donor for the sulfur atoms (Cicchillo et al., 2004; Harmer et al., 2014).

Lipoylation has mostly been studied in bacteria (reviewed in Cronan, 2016) and in *Escherichia coli*, two redundant pathways exist: *de novo* LA biosynthesis and scavenging of free lipoate. *De novo* biosynthesis is catalyzed by the sequential action of lipoyl synthase and octanoyltransferase (called LIPA and LIPB in bacteria: Morris et al., 1995). LIPB transfers the octanoyl chain from the octanoyl-ACP substrate to target proteins (Zhao et al., 2003, 2005) and then LIPA adds two sulfur atoms to generate LA. A second lipoylation pathway involves the activity of the lipoate protein ligase (LPLA) on free LA (Zhao et al., 2003). As such, *E. coli* LPLA attaches to free lipoate through a two-step ATP-dependent reaction in which lipoyl-AMP is an activated intermediate.

In plants, the 2-OGDH and glycine cleavage system complexes are exclusive to mitochondria, whereas PDH is also found in plastids where it is essential for *de novo* fatty acid biosynthesis (Martins-Noguerol et al., 2019). Both these organelles possess specific enzymatic machinery for LA biosynthesis and protein lipoylation. Plastidial lipoylation involves the concerted activities of LIP2 and LIP1 (Wada et al., 2001; Ewald et al., 2014). In mitochondria, lipoylation can take place through a similar pathway involving the LIP2 and LIP1 isoforms specific to that organelle (Wada et al., 2001), or by recycling free LA catalyzed by a LPLA similar to its bacterial homolog (Ewald et al., 2014).

The mitochondrial isoforms of LIP2 and LPLA have been characterized in *Arabidopsis thaliana* and they are essential for the plant’s viability (Ewald et al., 2014). In this regard, octanoyl-ACP for LIP2/LIP1 lipoylation is provided by β-oxidation or mitochondrial fatty acid synthase (FAS) activity, which in turn relies on a carbon supply in the form of acetyl-CoA produced by the lipoylated PDH complex. The crossed dependence of these processes makes lipoylation an important regulatory node for fatty acid biosynthesis in this organelle. In mitochondria, most of the *de novo* fatty acid synthesis is directed toward the synthesis of LA (Wada et al., 1997). Plastidial lipoyl synthases from sunflower have been characterized (Martins-Noguerol et al., 2020) and a set of plastidial octanoyltransferases has also been evaluated in this species. Here, a different system for LA synthesis in sunflower was found, comprised of a lipoyl synthase (*Ha*LIP1m) and an octanoyltransferase (*Ha*LIP2m) that differed in their sequence and predicted location from the aforementioned plastidial forms. Both genes were homologous to those previously described in *A. thaliana* mitochondria and they were both cloned, sequenced and characterized to study their expression and structure. The effect of the expression of these genes on fatty acid synthesis in *E. coli* and in transgenic Arabidopsis seeds, along with the description of the lipidomic remodeling in these transgenic seeds, illustrate the role of the mitochondrial lipoylation pathway in plant lipid metabolism.

## Materials and Methods

### Cloning of Two cDNAs That Encode the *Ha*LIP1m and *Ha*LIP2m Sequences

Sunflower genes homologous to those of *A. thaliana* mitochondrial lipoyl synthase (*AtLIP1m*; At2g20860) and octanoyltransferase (*AtLIP2m*; At1g04640) were identified by searching the Heliagene sunflower database^1^ (Badouin et al., 2017). Both genes were amplified from developing sunflower seed cDNA using specific pairs of primers containing the corresponding start and stop codons (HaLIP1m-F-BambHI, HaLIP1m-R-*Hin*dIII, HaLIP2m-F-BambHI, and HaLIP2m-R-*Hin*dIII: Supplementary Table 1). The resulting fragments were cloned into the pMBL-T Easy vector (Canvax, Spain) and examined by sequencing (Eurofins, Germany), the resulting BLASTp alignments confirming that they corresponded to complete ORFs containing the ATG and STOP codons. The sequences obtained were deposited in GenBank under accession numbers MT610108 (*HaLIP1m*) and MT610109 (*HaLIP2m*).

### Phylogenetic Characterization of *Ha*LIP1m and *Ha*LIP2m Protein Sequences

The deduced amino acid sequences for the *HaLIP1m* and *HaLIP2m* genes were aligned with homologous sequences using the ClustalX v.2.0.10 program (Larkin et al., 2007), employing the default settings to produce the corresponding phylogenetic trees with the MEGA6 software tool (Tamura et al., 2013). This analysis assessed the support of three nodes by applying a bootstrap analysis with 10,000 replicates. Signal peptides were identified in the protein sequences using the network-based programs ESLPred (Bhasin and Raghava, 2004), iPSORT (Bannai et al., 2002), and DeepMito (Savojardo et al., 2020). *Ha*LIP1m and *Ha*LIP2m were aligned with homologous proteins from different phylogenetic groups using the ClustalX v.2.0.10 and BioEdit programs in order to study evolutionary conserved residues. For the *Ha*LIP1m alignment, the homologous proteins selected were from *Arabidopsis thaliana, Ricinus communis, Oryza sativa*, and *Physcomitrella patens*. For *Ha*LIP2m the species were *Arabidopsis thaliana, Ricinus communis, Oryza sativa*, and *Selaginella moellendorffii*. The location of critical residues involved in catalytic activity was determined by alignment with known and crystallized-structure of lipoyl synthases from *Mycobacterium tuberculosis* (McLaughlin et al., 2016) and *Thermosynechococcus elongatus* (Harmer et al., 2014) for *Ha*LIP1m, and for crystallized octanoyltransferase from *Mycobacterium tuberculosis* (Ma et al., 2006) for *Ha*LIP2m.

### Modeling Three-Dimensional Structures and Molecular Docking

The Swiss Model Server (Schwede et al., 2003^2^) was used for structural modeling and the tertiary structures of *Ha*LIP1m and *Ha*LIP2m were modeled using available homologous X-ray structures as templates: lipoyl synthase from *Mycobacterium tuberculosis* (*Mt*LIPA, Protein Data Bank Accession 5EXK: McLaughlin et al., 2016) and octanoyltransferase from *Mycobacterium tuberculosis* (*Mt*LIPB, Protein Data Bank Accession 2QHS: Ma et al., 2006). Furthermore, molecular docking models were obtained for both novel proteins using the SwissDock server (Grosdidier et al., 2011). The substrates for docking were lipoyl-Lys (ZINC12494640) and 5′-deoxyadenosine radical (ZINC01999286) for *Ha*LIP1m, and octanoic acid (ZINC01530416) for *Ha*LIP2m. The models were visualized with the UCSF Chimera program (Pettersen et al., 2004).

### Expression in Sunflower Tissues

The expression of *HaLIP1m* and *HaLIP2m* was assessed by RT-qPCR using cDNAs from different vegetative tissues (roots, stems, cotyledons, and leaves) and developing seeds (12, 14, 18, 20, 25, and 28 days after flowering – DAF), and with primer pairs specific to each gene (HaLIP1m-qpcr-F and HaLIP1m-qpcr-R for *HaLIP1m* and HaLIP2m-qpcr-F and HaLIP2m-qpcr-R for *HaLIP2m*: Supplementary Table 1). The reactions were performed with SYBR Green I (QuantiTect SYBR Green PCR Kit, Qiagen, United Kingdom) over 50 PCR cycles of: 94°C for 30 s, 57°C for 30 s, and 72°C for 1 min. The expression of the sunflower actin gene *HaACT1* (GenBank Accession FJ487620) was assessed with the HaActin-qpcr-F4 and HaActin-qpcr-R4 primers (Supplementary Table 1) to normalize the data. The relative expression of the genes was calculated using the 2^–ΔΔ*CT*^ method.

### Expression and Purification of Recombinant Proteins in *Escherichia coli*

Sequences corresponding to mature *Ha*LIP1m and *Ha*LIP2m were cloned into the pQE-80L expression vector (Qiagen, Germany) using the *Bam*HI/*Hin*dIII restriction sites to produce proteins fused to a N-terminal (His)_6_-tag for purification. PCR amplicons of both genes were digested with *Bam*HI and *Hin*dIII, and ligated into the opened pQE-80L vector to produce the pQE-80L:*HaLIP1m* and pQE-80L:*HaLIP2m* constructs, later confirmed by sequencing. The *E. coli* XL1-Blue strain was transformed with these vectors and cultures were grown at 37°C in LB media (1% Bacto Tryptone, 0.5% yeast extract and 1% NaCl [pH 7.0]) supplemented with 50 μg/mL ampicillin for plasmid selection. Isopropyl β-D-1-thiogalactopyranoside (IPTG) was added to a final concentration of 0.5 mM to induce protein expression when the cultures reached an OD_600 nm_ value of 0.4. After induction, the cultures were grown for 4 h and the cells were harvested by centrifugation at 3000 × *g* for 20 min. The pellets were resuspended in Binding Buffer (20 mM sodium phosphate [pH 7.4], 500 mM NaCl and 20 mM imidazole) and the cells were then disrupted by 15 cycles of sonication at 70° amplitude during 10 s pulses with 10 s intervals for cooling on ice. The lysate was centrifuged at 2000 × *g* at 4°C for 20 min to recover the clear supernatant with the soluble fraction for protein purification. Proteins were purified using the His SpinTrap Kit (GE Healthcare, United Kingdom) according to the manufacturer instructions. Eluates obtained at different imidazole concentrations (100–500 mM) were collected and submitted to SDS-PAGE electrophoresis to monitor the presence of the recombinant protein (see Martins-Noguerol et al., 2020).

### Total Fatty Acid Composition of *Escherichia coli*

*Escherichia coli* cultures harboring pQE-80L:*HaLIP1m* and pQE-80L:*HaLIP2m* were grown at 37°C with 220 rpm shaking, inducing protein expression at an OD_600 nm_ of 0.4. After induction, the cultures were grown for 4 h and the cells were then recovered by centrifugation at 3000 × *g* for 20 min, washing the pellets twice with sterilized water. Cultures harboring the empty pQE-80L vector were used as controls. The total lipids from the cultures were methylated by adding 3 mL of the methylation mixture containing methanol/toluene/sulfuric acid (88:10:2, v/v/v) and by heating at 80°C for 1 h (Garcés and Mancha, 1993), and heptadecanoic acid (17:0, 150 μg) was added to the samples as an internal standard. Total fatty acids methyl esters (FAMES) were extracted with 1 mL hexane, transferred to fresh tubes and washed with 2 mL Na_2_SO_4_ (6.7%). Again, the upper phase was recovered in clean tubes and the solvent was evaporated under nitrogen. Methyl esters were finally resuspended in 200 μL heptane for gas chromatography (GC) analysis as described previously (González-Mellado et al., 2010).

### Functional Complementation of an *Escherichia coli lipA* Strain

To verify the activity of *Ha*LIP1m protein *in vivo* genetic complementation of an *E. coli* strain lacking lipoyl synthase (*E. coli lipA*, JW0623) was performed, a strain obtained from the Coli Genetic Stock Center (CGSC). Electrocompetent cells were generated and then transformed by electroporation with the pQE-80L:*HaLIP1m* or the empty pQE-80L vector. Transformants were incubated at 37°C overnight (ON) on LB-agar media plates supplemented with kanamycin (30 μg/mL) and ampicillin (50 μg/mL) for selection, and the resulting colonies were inoculated in 30 mL M9 glucose minimal medium and grown at 37°C ON. Subsequently, 5 mL of these cultures at 0.1 OD_600 nm_ were used to inoculate M9 glucose medium supplemented with ampicillin, kanamycin and 0.5 mM IPTG for plasmid induction. The JW0623 mutant transformed with empty pQE-80L, with and without LA supplementation (50 ng/mL), was used as a positive and negative control, respectively. The OD_600 nm_ was monitored every 90 min over 9 h, and OD_600 nm_ measurements were taken after 24 and 30 h.

### Generation of Transgenic *Arabidopsis thaliana* Plants

The complete gene sequences for both *HaLIP1m* and *HaLIP2m* were cloned into the pBIN19-35S binary vector driven by the 35S promoter from cauliflower mosaic virus (CaMV). PCR reactions with specific primers were performed to produce the gene sequences flanked by the *Bam*HI and *Hin*dIII restriction sites (HaLIP1m-F-*Bam*HI and HaLIP1m-R-*Hin*dIII for *HaLIP1m*/HaLIP2m-F-*Bam*HI and HaLIP2m-R-*Xba*I for *HaLIP2m*: Supplementary Table 1). A specific pair of primers for the pBIN19-35S vector were designed to screen the CAMV-35S-F and pBIN19-R constructs (Supplementary Table 1), which were subsequently confirmed by sequencing. Then constructs were then transformed into *Agrobacterium tumefaciens* strain GV3101 competent cells.

*Arabidopsis thaliana* Columbia-0 (Col-0) ecotype plants were grown in a growth chamber under a controlled environment (22°C day/20°C night, 60% humidity, 16-h, 250 μmol m^–2^ s^–1^ photoperiod). Transgenic Arabidopsis lines were generated by floral dip transformation as described previously (Sayanova et al., 2006). First generation seeds from the transformed plants were selected by germination in MS medium supplemented with kanamycin (50 μg/mL: as described in Harrison et al., 2006). Genomic DNA extracted from leaves of the growing plants was used to confirm the insertion of the sunflower gene into the *A. thaliana* genome by PCR. In the positive transgenic plants, the expression of both *HaLIP1m* and *HaLIP2m* was confirmed by RT-PCR using HaLIP1m-F/HaLIP1m-R, HaLIP2m-F-*Bam*HI/HaLIP2m-R-*Hin*dIII and At18S-F/At18S-R pair of primers (Supplementary Table 1). Third generation seeds of confirmed transgenic *A. thaliana* plants were used to analyze their lipid composition.

### Fatty Acids Analysis of Transgenic *Arabidopsis thaliana* Seeds

The fatty acid composition of the total lipid seed extract was determined as described previously (Martins-Noguerol et al., 2020). Three replicates were prepared using 50 seeds for each genotype (transgenic *HaLIP1m*, transgenic *HaLIP2m* and wild type, WT), which were ground in 3 mL hexane:isopropanol 3:2 (v/v). The samples were the mixed with 1.5 mL of 6.7% Na_2_SO_4_ (w/v) and the organic upper phase was transferred into new tubes. The aqueous residue was re-extracted with 2.5 mL hexane:isopropanol 7:2 (v/v) and the upper phase obtained was combined with the organic phase collected previously. The solvent was then evaporated under nitrogen and the lipids obtained resuspended in 200 μL heptane. Methylation of lipids was performed by adding 2 mL of the methylation mixture (methanol/toluene/H_2_SO_4_, 88:10:2 v/v/v) and maintaining this at 80°C for 1 h, before adding 2 mL hexane and recovering the upper phase. The organic phase was then washed with 1.5 mL of 6.7% Na_2_SO_4_ (w/v), the solvent was evaporated under nitrogen, and the precipitate containing the FAMES was resuspended in 200 μL heptane and analyzed by GC on a Hewlett Packard 6890 gas chromatograph (Palo Alto, CA, United States) with a Supelco SP-2380 fused-silica capillary column (30 m length, 0.25 mm i.d., 0.20 mm film thickness: Supelco, Bellefonte, PA, United States).

### Lipidomic Analysis of Transgenic Seeds

For lipidomic studies, lipids were extracted from 20 mg of ice-dried Arabidopsis seeds as described previously (Martins-Noguerol et al., 2019). The solvent was evaporated under an atmosphere of nitrogen and the lipids were solubilized in 200 μL isopropanol. The samples were then diluted fourfold and analyzed by ultra-high performance liquid chromatography coupled with quadrupole-time of flight mass spectrometry (LC-HRMS2) following the protocol described elsewhere with some modifications (Ulmer et al., 2017). LC was performed by HPLC 1290 (Agilent Technologies) and the lipid species were separated on a C18 Hypersil Gold column (100 × 2.1 mm, 1.9 μm. Thermofisher) following the temperature and gradient solvent conditions described (Martins-Noguerol et al., 2019). LC-electrospray ionization (ESI)-HRMS2 analysis was performed by coupling the LC system to a hybrid quadrupole time-of-flight high definition (QToF) mass spectrometer (Agilent 6538, Agilent Technologies) equipped with an ESI dual source. MassHunter B.07 software was used to control the parameters and the chromatogram was built as indicated (Martins-Noguerol et al., 2020). The peaks were annotated using two different databases: lipid Match (Koelmel et al., 2017) and lipid Blast (Kind et al., 2013). The normalization was performed by the sum of all the lipid species annotated (peak height annotated^∗^100/Sum of all peaks annotated).

### Statistical Analysis

All the statistical analyses were performed with the IBM SPSS v. 24.0 software (IBM Corp., Armonk, NY, United States). The data were tested for normality (Kolmogorov–Smirnov test) and for homogeneity of variance (Levene test), and then a one-way analysis of variance (ANOVA) was performed and significant differences were determined with the SNK test. The data from the lipidomic analysis were analyzed using Metaboanalyst v4.0 (Chong et al., 2019), performing a multivariant analysis. A Principal Component Analysis (PCA) was performed to study the differences in the lipid profiles among the different genotypes and an agglomerative analysis was then carried out to define hierarchical clusters. These clusters were represented in heatmaps where cells represent the concentration of each lipid species.

## Results and Discussion

LIP1 and LIP2 are the enzymes involved in *de novo* LA biosynthesis, the former an essential coenzyme for the activity of several enzyme complexes like PDH. Plants contains two distinct and spatially separated PDH complexes, one in the mitochondrial matrix that is the primary entry point for carbon into the citric acid cycle, and the other in the plastid stroma providing acetyl-coA for fatty acid biosynthesis. In plants, fatty acid biosynthesis mainly takes place in plastids and some of the acyl groups produced in that organelle are used as precursors for mitochondrial glycerolipids (Michaud et al., 2017). However, mitochondria also possess their own FAS, which differs from that of plastids (Wada et al., 1997; Gueguen et al., 2000; Guan and Nikolau, 2016; Guan et al., 2017; Fu et al., 2020). Unlike plastids, fatty acids are produced in mitochondria by a type II FAS, using malonate as a precursor. Among the metabolites synthesized by the mitochondrial pathways, octanoic acid has been demonstrated to be the major product (Wada et al., 1997), a molecule that is primarily used for the lipoylation of the target proteins.

### The Distribution and Phylogeny of Sunflower Octanoyltransferase and Lipoyl Synthase

A *LIP1* and *LIP2* gene homologous to the Arabidopsis mitochondrial genes were isolated here and cloned from sunflower seed cDNA (*HaLIP1m* and *HaLIP2m*). These genes were 1122 and 705 bp long, respectively, containing 372 amino acids (aa, *Ha*LIP1m) and 234 aa (*Ha*LIP2m). The sunflower genome contains two sets of genes related to *de novo* LA synthesis, one of which corresponds to the plastidial isoforms that support the synthesis of this co-factor and that contributes to the PDH complex in that organelle. These are nuclear genes with well-defined transit peptides, such as the plastidial lipoyl synthases characterized previously (Martins-Noguerol et al., 2020). Arabidopsis also possesses two sets of enzymes, one located in plastids (Yasuno and Wada, 1998; Ewald et al., 2014) and the other in mitochondria (Ewald et al., 2014), with the localization of LPLA and LIP2 in the latter confirmed in western blots of mitochondrial matrix proteins. These genes were necessary for plant viability as they support the synthesis and transfer of LA to mitochondrial complexes involved in essential functions for the plant, such as PDH.

Here, starting from the *Ha*LIP1m and *Ha*LIP2m sequences, we analyzed their distribution predicted by different bioinformatics platforms. In the case of *Ha*LIP1m, all platforms unequivocally identified it as a mitochondrial protein, with the Mitoprot, iPSORT, Predotar, PredSL, TPpred, MitoFates, and TargetP algorithms (reviewed by Martelli et al., 2021) yielding high scores for its location in the mitochondrial matrix (Supplementary Table 2). The hierarchical tree generated by the DeepLoc algorithm for protein location (Almagro Armenteros et al., 2017) also predicted a 99% probability of mitochondrial location for *Ha*LIP1m (Supplementary Figure 1). In the case of *Ha*LIP2m the predictions of the bioinformatics platforms were not so clear and while they yielded scores indicating a possible mitochondrial location, they were not conclusive (Supplementary Table 2). In this regard, the orthologous Arabidopsis enzyme, *At*LIP2m, yielded similar scores when launched in these prediction programs, the mitochondrial location of which has been demonstrated (Ewald et al., 2014). Hence, it seems that this family of proteins is not unequivocally identified as mitochondrial by the aforementioned algorithms. Considering that the presence of a complete LA synthesis system in the mitochondrial matrix is essential for the viability of plants, it would be expected that *Ha*LIP1m and *Ha*LIP2m form part of the mitochondrial lipoic synthesis system in sunflower, pending confirmation of this hypothesis through specific studies of their distribution.

Based on the sequences of these genes, both are nuclear encoded proteins with Nt mitochondria targeting sequence (MTS). Most mitochondrial proteins are encoded by nuclear genes and then transported into the target organelle in different ways (Hansen and Herrmann, 2019). The signal peptide of these proteins is an N-terminal sequence varying in length from 11 to 109 aa in *A. thaliana* (Zhang and Glaser, 2002). The transit peptide in *Ha*LIP1m had 69 aa with a predominance of hydroxylated (17.4% Ser and 13% Thr), hydrophobic (13% Leu) and positively charged (8.7% Arg) residues. *Ha*LIP2m had a 12 aa signal sequence with a predominance of Arg (25%, positively charged) and Leu (16.7%, hydrophobic) residues, while it also contained Glu, Ile, Met, and Val. There is strong variability in the amino acid composition of mitochondrial transit signals but with a prevalence of positively charged, hydroxylated and hydrophobic residues (Hansen and Herrmann, 2019), characteristics of the transit signals from the two novel sunflower proteins.

**FIGURE 1:**
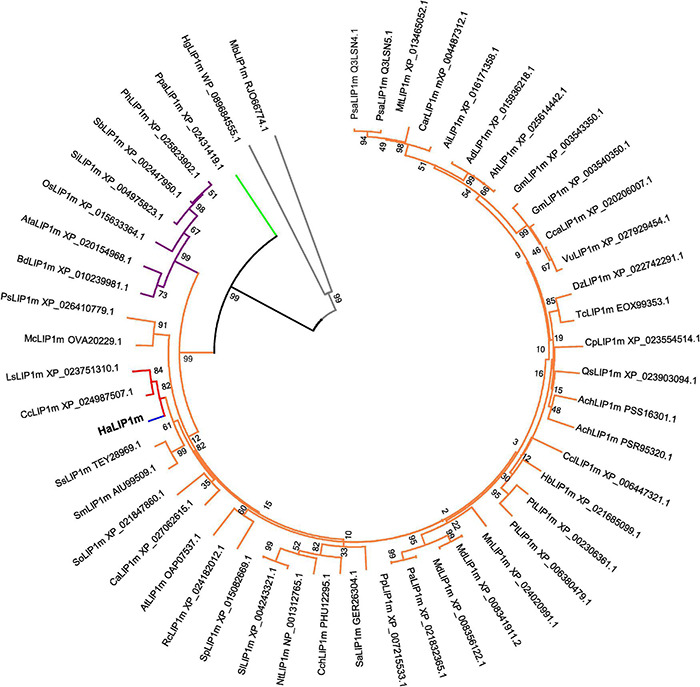
Phylogenetic tree of annotated mitochondrial lipoyl synthases. The plant species included in the tree are: *Ach, Actinidia chinensis; Ad, Arachis duranensis; Ah, Arachis hypogaea; Ai, Arachis ipaensis; At, Arabidopsis thaliana; Ata, Aegilops tauschii; Bd, Brachypodium distachyon; Ca, Coffea Arabica; Car, Cicer arietinum; Cc, Cynara cardunculus; Cca, Cajanus cajan; Cch, Capsicum chinense; Ccl, Citrus clementine; Cp, Cucurbita pepo; Dz, Durio zibethinus; Gm, Glycine max; Hb, Hevea brasiliensis; Ls, Lactuca sativa; Mc, Macleaya cordata; Md, Malus domestica; Mn, Morus notabilis; Mt, Medicago truncatula; Nt, Nicotiana tabacum; Os, Oryza sativa; Pa, Prunus avium; Ph, Panicum hallii; Pp, Prunus persica; Ps, Papaver somniferum; Psa, Pisum sativum; Pt, Populus trichocarpa; Qs, Quercus suber; Rc, Rosa chinensis; Sa, Striga asiatica; Sb, Sorghum bicolor; Si, Setaria italic; Sl, Solanum lycopersicum; Sm, Salvia miltiorrhiza; So, Spinacia oleracea; Sp, Solanum pennellii; Ss, Salvia splendens;Tc, Theobroma cacao;Vu, Vigna unguiculata.* LIP1m from a bryophyte species (*Ppa*. *Physcomitrella patens)* was used as the outgroup (green subtree). *Ha*LIP1m is marked in blue, the Asteraceae family in red, the Dicotyledoneae group in orange, Monocotyledoneae species in purple and two Proteobacteria species in gray (*Hg, Halomonas gudaonensis* and *Mb, Myxococcales bacterium*).

**FIGURE 2:**
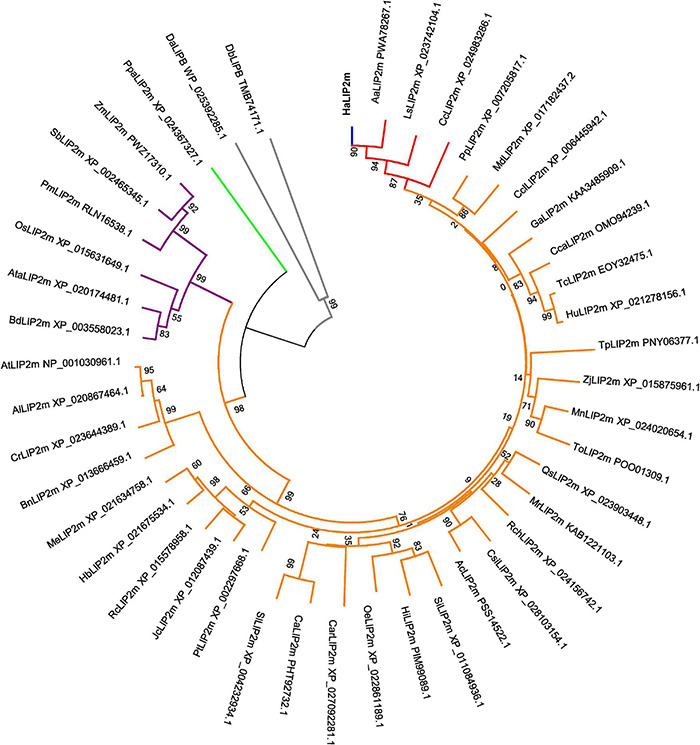
Phylogenetic tree of the annotated mitochondrial octanoyltransferases. Plant species included are: Aa, *Artemisia annua;* Ac, *Actinidia chinensis;* Al, *Arabidopsis lyrata;* At, *Arabidopsis thaliana;* Ata, *Aegilops tauschii;* Bd, *Brachypodium distachyon;* Bn, *Brassica napus;* Ca, *Capsicum annuum;* Car, *Coffea Arabica;* Cc, *Cynara cardunculus;* Cca, *Corchorus capsularis;* Ccl, *Citrus clementine;* Cr, *Capsella rubella;* Csi, *Camellia sinensis;* Ga, *Gossypium austral;* Ha, *Helianthus annuus;* Hb, *Hevea brasiliensis;* Hi, *Handroanthus impetiginosus;* Hu, *Herrania umbratica;* Jc, *Jatropha curcas;* Ls, *Lactuca sativa;* Md, *Malus domestica;* Me, *Manihot esculenta;* Mn, *Morus notabilis;* Mr, *Morella rubra;* Oe, *Olea europaea;* Os, *Oryza sativa*; Pm, *Panicum miliaceum;* Pp, *Prunus persica;* Pt, *Populus trichocarpa;* Qs, *Quercus suber;* Rc, *Ricinus communis;* Rch, *Rosa chinensis;* Sb, *Sorghum bicolor;* Si, *Sesamun indicum;* Tc, *Theobroma cacao;* To, *Trema orientale;* Tp, *Trifolium pratense;* Sl, *Solanum lycopersicum;* Zj, *Ziziphus jujuba;* Zm, *Zea mays.* LIP2m from a bryophyte (Ppa, *Physcomitrella patens)* was used as an outgroup (green subtree), and *Ha*LIP2m is marked in blue, the Asteraceae family in red the dicots in orange, the monocots in purple and two proteobacteria species in gray (Da, *Desulfurella acetivorans* and Db, *Deltaproteobacteria bacterium*).

A phylogenetic tree was generated that included the novel *Ha*LIP1m and *Ha*LIP2m proteins, and their known plant homologs (Figures 1, 2). In both dendrograms, *Ha*LIP1m and *Ha*LIP2m clustered together with the *Lactuca sativa* and *Cynara cardunculus* isoforms, close to the Solanaceae and Rosaceae families. These characteristics were also observed when the phylogeny of other sunflower proteins was studied (González-Thuillier et al., 2016; Troncoso-Ponce et al., 2018).

**FIGURE 3:**
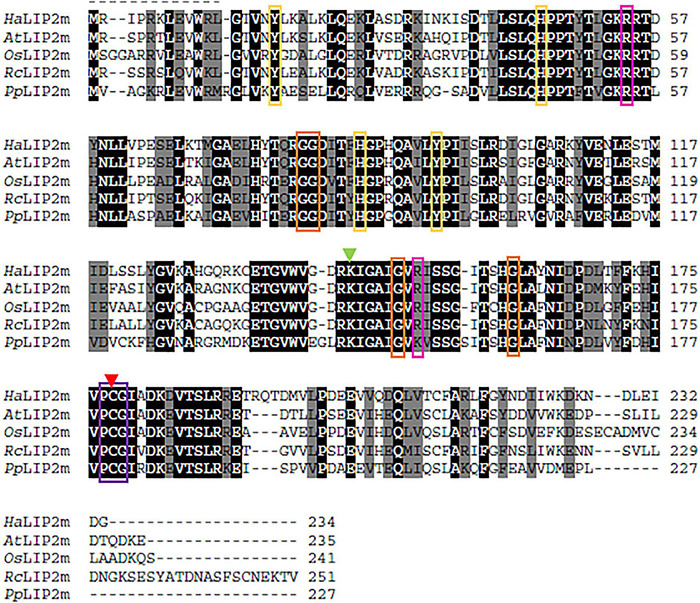
Alignment of the sunflower octanoyltransferase (*Ha*LIP2m) amino acid sequence with homologous sequences from species in different phylogenetic groups: *Arabidopsis thaliana* (*At*LIP2m; NP_001030961.1); *Ricinus communis* (*Rc*LIP2m; XP_015578958.1); *Oryza sativa* (*Os*LIP2m; XP_015631649.1); and *Physcomitrella patens* (*Pp*LIP2m; XP_024367327.1). Identical evolutionarily conserved residues are highlighted in black and highly conserved residues are highlighted in gray. Residues involved in substrate binding are indicated with a green (Lys) or red arrow (Cys). The conserved PCG motif is indicated by a purple box, while Gly residues involved in the formation of the cavity where the substrate is allocated are in orange boxes. Aromatic residues that presumably interact with the substrate are indicated by yellow boxes and other positively charged residues surrounding the access to the active site are in pink boxes.

**FIGURE 4:**
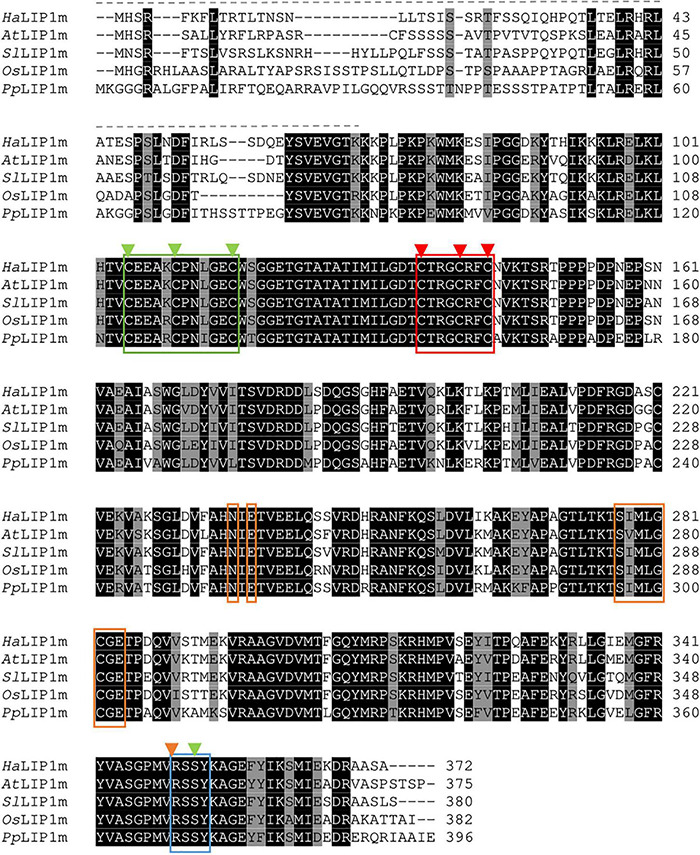
Alignment of the sunflower lipoyl synthase (*Ha*LIP1m) aa sequence with homologous proteins from *Arabidopsis thaliana* (*At*LIP1m; OAP07537.1); *Solanum lycopersicum* (*Sl*LIP1m; XP_004243321.1); *Oryza sativa* (*Os*LIP1m; XP_015633364.1); and *Physcomitrella patens* (*Pp*LIP1m; XP_024391419.1). Strictly conserved residues are highlighted in black and highly conserved residues in gray. The signal peptide sequence is indicated with a dashed line and the [4Fe–4S] clusters that participate in the catalytic activity of LIP1 are marked by a red (RS cluster involved in SAM cleavage) and green box (auxiliary cluster with the Ser residue that also coordinates this cluster marked with a green arrow). The conserved R(S/T)S motif in the C-terminus is marked by a blue box. The residues involved in SAM recognition are in orange boxes (Asn and Glu in the ribose motif, and the “GXIXGX_2_E” motif-like characteristic of RS enzymes) and with an orange arrow (Arg).

The protein alignments with homologs from different phylogenetic groups (Figures 3, 4) identified highly conserved domains, indicating a strong evolutionary conservation of these enzymes. The main differences were observed in the N- and C-termini of the proteins. The catalytic aa of both enzymes were found by aligning their sequences with *Mt*LIPA and *Mt*LIPB from *M. tuberculosis* (see Figures 3, 4), homologous proteins with a previously resolved structure (Ma et al., 2006; McLaughlin et al., 2016).

The activity of LIP2 first involves transfer of octanoate from acyl-ACP to form a thiol bond with a Cys residue in the enzyme, which is followed by the formation of an amide bond between the octanoate and a Lys residue of the target subunit, the E2 protein in the case of the PDH complex (Zhao et al., 2003; Ma et al., 2006). In the crystal structure of *Mt*LIPB, two invariant Cys and Lys residues are thought to act as acid-base catalysts for such transfers. The conserved Cys residue is also that which binds to octanoate during the reaction (Ma et al., 2006; Kim et al., 2008). These residues correspond to Cys178 and Lys144 in *Ha*LIP2m, and the Cys residue is part of a conserved PCG motif that is also found in *Ha*LIP2m (Pro177-Cys1778-Gly179: Figure 3). Several aromatic residues are also thought to be involved in the substrate interaction of *Mt*LIPB and they form a hydrophobic pocket that interacts with the substrate (Tyr22, His49, His83, and Tyr91: Ma et al., 2006), the homologs of which in *Ha*LIP2m were Tyr17, His45, His86, Tyr94 (Figure 3). Moreover, other Gly residues are involved in the formation of the cavity depicted in *Mt*LIPB, these residues corresponding to Gly80, Gly81, Gly149, and Gly160 in *Ha*LIP2m (Figure 3). Like other enzymes depending on acyl-ACP, several positive charged residues are detected at the substrate access site, such as Arg58, Arg130, and Arg149 in *Mt*LIPB (Ma et al., 2006). Two of these homologous residues have been detected in *Ha*LIP2m (Arg54 and Arg151), although one Arg residue was unexpectedly replaced by a negatively charged one (Glu135). However, this Glu was also found in this position in other plant LIP1m sequences used in the alignment (see Figure 3).

The reaction catalyzed by LIP1 involves the insertion of two sulfur atoms into an octanoyl-chain bound to the apo-subunit to form LA in a reaction that depends on SAM as a co-factor. Two invariant 4[Fe–4S] clusters are present in this protein, one of which is bound by three conserved Cys residues that constitute the CX_3_CX_2_C motif, a characteristic of proteins that reduce SAM (this cluster is referred to as the “RS cluster”). In *Ha*LIP1m this RS cluster is bound by Cys136, Cys140, and Cys 143 (boxed in red in Figure 4). The second or “auxiliary” cluster is specific to lipoyl synthases and it is coordinated by a CX_3_CX_2_C motif. Accordingly, this cluster is coordinated by Cys105, Cys110, and Cys116 in *Ha*LIP1m (indicated in green in Figure 4). This auxiliary cluster also binds to a highly unusual Ser residue located close to the C-terminus and that is conserved in LIP1 enzymes, corresponding to Ser352 in *Ha*LIP1m (in green in Figure 4). Other residues characteristic of the RS protein superfamily were also identified in *Ha*LIP1m, including: (i) two invariant Asn and Glu residues that correspond to Asn233 and Glu238 in *Ha*LIP1m (orange boxes in Figure 4); (ii) a “GXIXGX_2_E” motif corresponding to S_277_IMLGCGE_285_ in *Ha*LIP1m (blue box in Figure 4); and (iii) a conserved Met residue, Met279 in *Ha*LIP1m (marked with a blue arrow as part of the “GXIXGX_2_E” motif in Figure 4).

### Tertiary Structure Prediction and Molecular Docking

**FIGURE 5:**
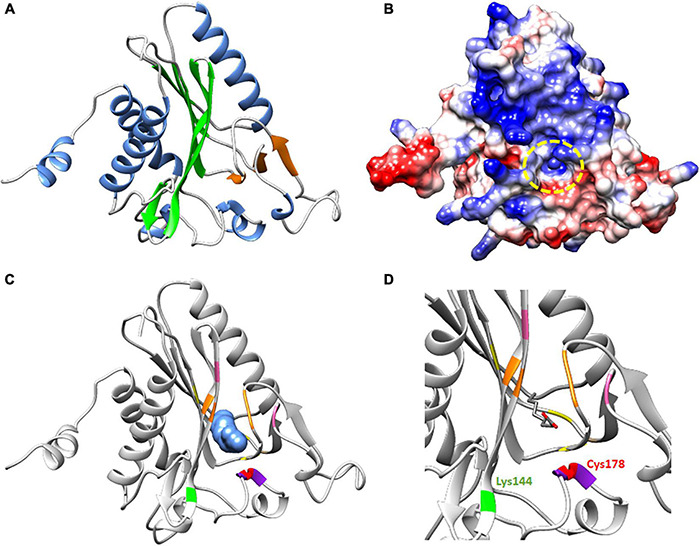
Proposed structure and molecular docking model for *Ha*LIP2m. **(A)** Tertiary structure. The core consisted of two β sheets (a minor β sheet in orange and a large β sheet in green). The secondary structure elements with α helix motif are in blue. **(B)** Coulombic surface coloring showing the gap where the octanoyl chain interacts. **(C)** Ribbon diagram with the octanoic acid substrate in a 3D view next to the Cys residue (in red and forming part of the purple PCG motif). The Lys residue involved in acyltransferase activity with Cys/Lys is in green and the conserved Gly residues forming the gap in the core structure are in orange. The positively charged residues that participate in the interaction with substrate are marked in pink and the aromatic residues also involved in the interaction with the substrate are in yellow. **(D)** Wider angle of the active center of the protein with the substrate in a ribbon diagram.

A structural model of *Ha*LIP2m was obtain based on the crystal structure of *Mt*LIPB (Ma et al., 2006) as *Ha*LIP2m and *Mt*LIPB share 45.90% of identity. The model consisted of a single monomer constituted by eight α helices and nine β strands, with the β strands grouped into two β sheets with a gap between both (Figures 5A,B). The larger β sheet contained six β strands (β1β2-β6-β7-β8-β9) and the minor β sheet was formed by three β strands (β3-β4-β5). LIP2 proceeds by transferring the acyl group from an octanoyl-ACP substrate to a specific Lys residue in the target apo-proteins. For this activity, LIP2 forms a thioester intermediate with an octanoyl chain via the conserved Cys residue in the structure (Ma et al., 2006; Kim et al., 2008). The docking model of *Ha*LIP2m using octanoic acid as a substrate (Figures 5B–D) was consistent with this hypothesis, and in our docking model octanoic acid was positioned in the core gap between the two β sheets, with the carboxylic group outside and next to Cys178. This Cys187 also formed part of the conserved PCG motif and it lay next to the conserved Lys144 residue. To transfer the acyl group from the octanoyl-ACP substrate to the specific Lys residue in the target apo-proteins, LIP2 forms a thioester intermediate with the octanoyl chain through the conserved Cys residue in the structure (Zhao et al., 2005; Ma et al., 2006). Our docking model is consistent with this hypothesis and we propose that Cys178 is responsible for the thioester bond that forms with the octanoyl chain to establish this intermediate during catalysis, as this Cys178 remains next to the carboxyl group (Figure 5D). The conserved Lys residue probably interacts with this Cys residue when a substrate is not available (Ma et al., 2006). Accordingly, the conserved Lys144 in *Ha*LIP2m remains next to Cys178 in the 3D structure (Figure 5D), which would be consistent with its proposed function. Other studies have provided evidence for this mechanism of action. The structure of LIPB from *Thermus thermophilus* (*Tth*LIPB: Kim et al., 2008), which has been crystallized with decanoic acid, revealed the same Cys and Lys residues to be involved in catalysis. In addition, the activity of LIPB from *E. coli* (*Ec*LIPB) appears to proceed by forming a thioester intermediate with the thiol group from a conserved Cys residue (Cys169 in *Ec*LIPB: Zhao et al., 2005).

**FIGURE 6:**
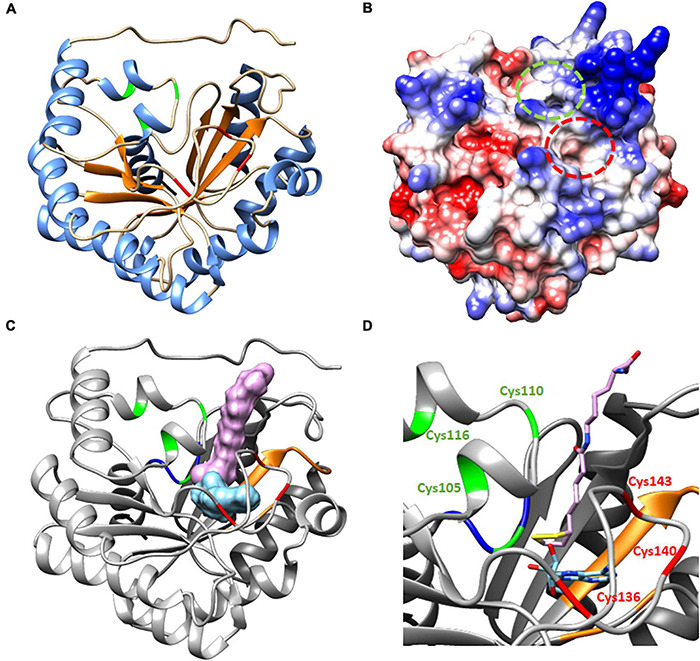
Proposed structure and molecular docking model for *Ha*LIP1m. **(A)** TIM partial barrel structure showing the secondary structure elements, with an α helix motif are in blue and the β strands are in orange. **(B)** Coulombic surface coloring showing the gaps where substrates interact. Indicated in red is the gap where octanoyl-Lys is positioned to form lipoyl-Lys, while the gap where SAM interacts to form 5′-deoxyadenosyne radicals is in green. **(C)** Molecular docking. 3D Ribbon diagram with substrates (lipoyl-lys and 5′deoxiadenosine) located at the active site of protein. The residues involved in catalytic activity are in red (the RS cluster responsible for SAM reduction) and green (auxiliary cluster donating the sulfur atoms during LA synthesis). The Ser residue in green forms part of the R(S/T)Y motif (in blue) located close to the auxiliary cluster that allows the coordination of the cluster. The residues involved in SAM recognition are in orange and the lipoyl-lys peptide chain is positioned out of the gap with the sulfur atoms of the LA ring toward the core of the protein, close to 5′deoxiadenosine. **(D)** Amplified ribbon diagram of the active center of the protein with the substrates.

A structural model of *Ha*LIP1m was obtained based on the crystal structure of *Mt*LIPA (McLaughlin et al., 2016), as both these proteins sharing 41.26% identity (see alignments in Supplementary Figure 2). The model obtained had a monomeric structure consisting of 13 α helices and 7 β strands (β7/α13), and there was a partial TIM barrel pattern (Figure 6A) described previously for other lipoyl synthases, even though the number of α helix or β strands varies (Harmer et al., 2014; McLaughlin et al., 2016). In this model, the β strands remained in the core of the protein while the α helices had an outward disposition. This was a structure very similar to the two plastidial isoforms of sunflower lipoyl synthase (*Ha*LIP1p1 and *Ha*LIP1p2) characterized previously by our group (Martins-Noguerol et al., 2020).

Two gaps were visible in the computed surface model of *Ha*LIP1m (Figure 6B). In the docking model, these gaps were occupied by the substrates selected, the peptide chain of the lipoyl-lys substrate remained outside the gap while the sulfur atoms were situated within the structure, 5′-deoxyadenosine staying close to the lipoyl group (Figures 6C,D). The lipoyl-lys substrate is positioned near to the auxiliary cluster and it is responsible for the insertion of sulfur into the octanoyl chain and the generation of lipoyl-lys. In addition, both docking substrates were located very close together, with the sulfur atoms of lipoyl-lys toward the core of the structure. The unusual Ser residue that coordinates the auxiliary cluster was also located very close to this cluster at the active site of the protein. The 5′-deoxyadenosine was also located near to the RS cluster responsible for the cleavage of SAM and the generation of the radical. Site-directed mutagenesis suggests that the Ser residue is essential for lipoyl group formation but not for the reductive cleavage of SAM (Harmer et al., 2014). Moreover, alignment of 590 lipoyl synthase sequences (Akiva et al., 2014) shows that the serine ligand is conserved in over 98% of these sequences. These findings are consistent with the mechanism of action described for lipoyl synthases. When the substrate is available, the octanoyl chain bound to the target apo-protein is positioned close to the N-terminus of lipoyl synthase and this chain is accommodated between the two [4Fe–4S] clusters (McLaughlin et al., 2016). A conformational change of the structure has then been described that bring the clusters together, involving a shortening from 15.3 to 11.8 Å in *Mt*LIPA (McLaughlin et al., 2016). The docking model of *Ha*LIP1m reflected the structure of the protein when this conformational change took place (Figure 6) as the reference model is a structure crystallized with the substrate. The proposed conformational change enables the reduction of SAM and the first sulfur insertion of the octanoyl chain at C6 through the action of the RS cluster. The cleavage of SAM generates methionine and a 5′-deoxyadenosyl radical. Although the exact function of the auxiliary cluster has not yet been fully established it has been suggested that both sulfur atoms are transferred from a single lipoyl synthase molecule (Cicchillo et al., 2004). Accordingly, the auxiliary cluster acts as the sulfur donor for the second insertion at C8. This hypothesis involves the scarification of the auxiliary cluster during activity (Cronan, 2016), although this mechanism remains controversial.

### *HaLIP1m* and *HaLIP2m* Expression

**FIGURE 7:**
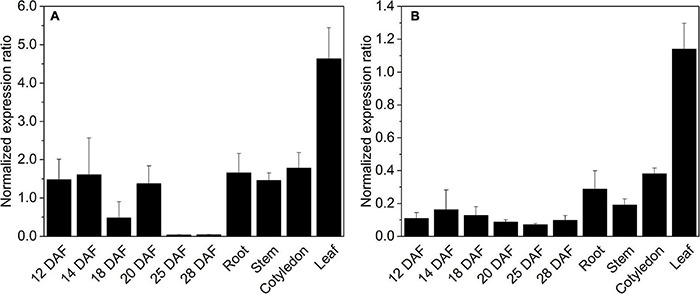
Expression of octanoyltransferase *HaLIP1m* **(A)** and lipoyl synthase *HaLIP2m* **(B)** in *Helianthus annuus* seeds at different developmental stages and in distinct tissues. Gene expression was normalized to the expression of *HaACT1*: DAF, days after flowering. The data represent the average values ± SD of three independent samples.

The expression of *HaLIP1m* and *HaLIP2m* was analyzed by RT-qPCR in different vegetative sunflower tissues and in developing seeds. Transcripts from these genes accumulated in all the tissues analyzed, with the expression of *HaLIP2m* always several fold-higher than that of *HaLIP1m*. Both genes were maximally expressed in leaf tissue (Figure 7) and both genes were expressed similarly in developing sunflower seeds, with a decrease in the number of transcripts at later developmental stages (25–28 DAF: Figure 7). Therefore, plant leaf mesophyll tissue has a high LA requirement as these cells have large amounts of the glycine cleavage system complex to catalyze the conversion of Gly into Ser during photorespiration (Bauwe et al., 2010). Hence, leaf mesophyll is the main site of LA biosynthesis in plants (Wada et al., 1997) and consequently *HaLIP1m* and *HaLIP2m* were expressed most strongly in these tissues. By contrast, LIP2m from *A. thaliana* has been described as an essential protein expressed mostly in leaves and photosynthetic organs, yet not in roots and flowers (Ewald et al., 2014).

### Complementation of JW0623 With the Sunflower *HaLIP1m* Gene

**FIGURE 8:**
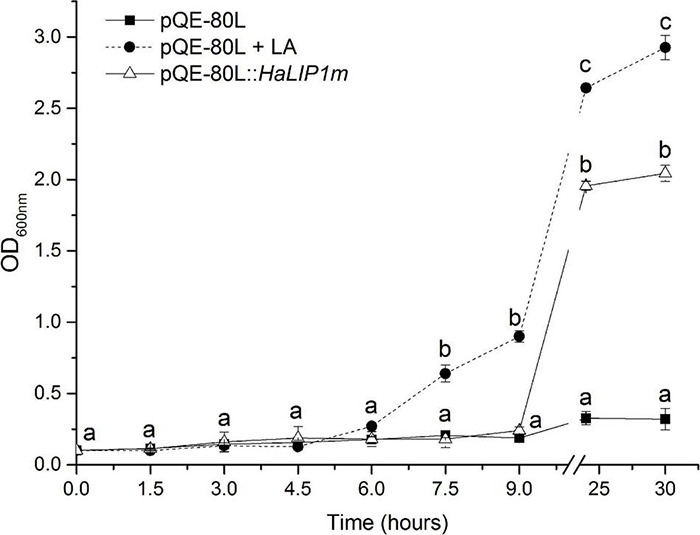
Complementation of LipA-deficient JW0623 *E. coli* in M9 glucose minimal medium following transformation with *HaLIP1m* in a pQE-80L expression vector, and using *E. coli* cells transformed with the empty pQE-80L vector as a control: LA, lipoic acid supplementation (50 ng/mL). All cultures were induced with 0.5 mM IPTG and the break in the graph separates the growth during the first 9 h from the measurements at 24 and 30 h. The data represent the mean ± SD of three independent replicates and the different letters are the mean statistical differences (*p* < 0.05) at each time point.

In order to ratify the activity of *HaLIP1m in vivo*, a genetic complementation study was performed in the JW0623 *lipA* deficient *E. coli* mutant strain that grows poorly even in rich medium (Santos and Hirshfield, 2016). The growth rate of this stain was even slower in M9 minimal medium where no free LA is available for lipoate scavenging by LPLA. *HaLIP1m* expression was able to restore the normal growth rate of the mutant (Figure 8). Although in the first hours of *HaLIP1m* expression by the mutant it grew in parallel to the negative control (*E. coli* mutant strain transfected with the empty vector), a normal growth rate was recovered after 24 h similar to that of the positive control (*E. coli* with empty vector in minimal medium supplemented with LA). Hence, the *HaLIP1m* expressed by bacteria clearly produced a functional enzyme.

### Fatty Acid Analysis of *Escherichia coli* Expressing *HaLIP1m* and *HaLIP2m*

The mature protein coding regions of both *HaLIP1m* and *HaLIP2m* were cloned into pQE-80L expression vector to express these genes heterologously in *E. coli* XL1-Blue cells. Both proteins were expressed strongly in this system and they were purified from the soluble fraction of the cell lysates by affinity chromatography on Ni-NTA columns as recombinant proteins of the predicted molecular weight (Supplementary Figure 3). Qualitative modifications in terms of bacterial fatty acid composition were measured, and overexpression of both *HaLIP1m* and *HaLIP2m* produced changes in some fatty acid species relative to the fatty acids isolated from the controls carrying the empty pQE-80L vector (Table 1). Although these changes were quite mild, significantly *HaLIP1m* produced an increase of 18:1^Δ^ ^11^ while *HaLIP2m* increased the 18:1^Δ^ ^11^ species and decreased the 14:0, 16:0, and 19:0Δ species. Moreover, *HaLIP2m* overexpression produced an increase in the unsaturated to saturated fatty acid ratio (UFA/SFA; Table 1) and a decrease in the total amount of fatty acids was detected in both strains overexpressing the heterologous sunflower proteins (Table 1). In *E. coli*, LA is synthesized from octanoyl-ACP through the sequential activity of LIPB and LIPA, although octanoyl-ACP can also be used by β-ketoacyl-ACP synthase I (FABB in *E. coli*) for acyl chain elongation to form decanoic acid. The data from *E. coli* expressing *HaLIP2m* could be explained by competition between this enzyme and LIPB or FABB for the intracellular octanoyl-ACP substrate. Thus, the overexpression of *HaLIP2m* could produce octanoyl-ACP depletion (which would remain bound to *Ha*LIP2m to form the acyl thioester intermediate), reducing its availability for the synthesis of longer fatty acids and inducing the decrease in fatty acid synthesis observed. Furthermore, a decrease in the growth of bacteria harboring *HaLIP2m* was observed (data not shown) which supports the competition hypothesis.

**TABLE 1:** Fatty acid composition (mol%) of *E. coli* transformed with recombinant plasmids.

	**pQE-80L**	**pQE-80L*:HaLIP1m***	**pQE-80L*:HaLIP2m***
**14:0**	3.75 ± 0.35**^a^**	3.80 ± 0.26**^a^**	2.81 ± 0.29**^b^**
16:0	45.00 ± 0.49^a^	46.32 ± 1.90^a^	42.30 ± 0.35^b^
16:1	20.32 ± 3.07^a^	22.60 ± 0.69^a^	16.52 ± 0.52^a^
17:0Δ^a^	9.94 ± 2.78^a^	6.01 ± 3.32^a^	10.41 ± 0.55^a^
18:0	0.94 ± 0.21^a^	0.75 ± 0.02^a^	1.63 ± 0.06^a^
18:1^**Δ**11^	18.95 ± 0.34^a^	20.26 ± 0.64^b^	25.68 ± 0.34^b^
19:0Δ^a^	1.10 ± 0.69^a^	0.26 ± 0.15^a^	0.65 ± 0.06^b^
SFA^b^	49.69 ± 0.31^a^	50.88 ± 2.13^a^	46.74 ± 0.06^b^
UFA^*c*^	50.31 ± 0.31^a^	49.12 ± 2.13^a^	53.26 ± 0.60^b^
UFA/SFA	1.01 ± 0.01^a^	0.97 ± 0.08^a^	1.14 ± 0.03^b^
mg FAs/unit OD600 nm	0.44 ± 0.09^a^	0.36 ± 0.01^ab^	0.30 ± 0.06^b^

*Fatty acids: 14:0, myristic acid; 16:0, palmitic acid; 16:1Δ9, palmitoleic acid; 17:0Δ, *cis*-9,10-methylenehexadecanoic acid; 18:0, stearic acid; 18:1Δ11, *cis*-vaccenic acid; 19:0Δ, *cis*-9,10-methyleneoctadecanoic acid. Protein expression was induced with 0.5 mM IPTG, and the data represent the average ± SD of three independent samples (*HaLIP1m* and *HaLIP2m*) and of six samples for the control cultures transformed with the empty pQE-80L. Different letters represent statistical differences (*p* < 0.05).*

*^a^C17 and 19 cyclopropanes derived from C16:1 and 18:1, respectively.*

*^*b*^Saturated fatty acids 14:0 + 16:0 + 18:0.*

*^*c*^Unsaturated fatty acids and their derivatives 16:1^Δ9^ + 17:0Δ + 18:1^Δ11^ + 19:0Δ.*

The fatty acid profile associated with *HaLIP1m* was essentially the same as in the control. Overexpression of lipoyl synthase activity in bacteria was expected to produce more LA and more lipoylation of the E2-PDH complex, enhancing its activity and consequently, more acetyl-CoA can be produced for fatty acid biosynthesis. However, there were no significant differences in the total amount of fatty acids in the transgenic cultures relative to the controls. This could reflect the endogenous regulation of PDH by bacterial lipoylation machinery to maintain its activity.

Together these results indicated that both the *Ha*LIP2m and *Ha*LIP1m enzymes are functional and that they can modify fatty acid metabolism in bacteria. Other target complexes for LA in bacteria are 2-OGDH, which acts in the citric acid cycle for cell respiration, and the glycine cleavage system, yielding carbon dioxide, ammonia, 5,10-methylenetetrahydrofolate plus a reduced pyridine nucleotide (Cronan et al., 2005). In this context, it is important to consider that *Ha*LIP2m and *Ha*LIP1m could also interact with these other metabolic pathways.

### Effect of *HaLIP1m* and *HaLIP2m* Expression on the *Arabidopsis thaliana* Lipidome

Most plant fatty acids are synthetized in plastids (or chloroplasts in green tissues) and they serve as precursors for glycolipids (the main lipids forming photosynthetic membranes), phospholipids (the principal components of cell membranes) or acylglycerols (including triacylglycerols – TAGs-, the main compounds in storage lipid seeds). Mitochondria also have their own fatty acid biosynthetic machinery, which is mainly dedicated to produce LA and lipid A (Guan et al., 2017). Although, the majority of fatty acids for lipid biosynthesis are derived from intraplastidial biosynthesis, there is evidence that fatty acid biosynthesis in mitochondria is essential for correct plant lipid metabolism (Guan et al., 2017). Transgenic *A. thaliana* plants overexpressing *HaLIP1m* or *HaLIP2m* did not show any external phenotype (Supplementary Figure 4) and thus, 3rd generation of transgenic Arabidopsis seeds, confirmed by RT-PCR (Supplementary Figure 5), were used to analyze the total fatty acid composition of seeds (Table 2). No differences in seed fatty acid composition were observed when *HaLIP2m* was overexpressed. However, some minor changes were found in seeds when they overexpressed *HaLIP1m*, in particular a mild increase in linolenic acid at the expense of oleic and linoleic acid (Table 2).

**TABLE 2:** Fatty acid composition (mol%) of *A. thaliana* seeds overexpressing *HaLIP1m* or *HaLIP2m*.

	**WT Col-0**	***HaLIP1m***	***HaLIP2m***
16:0	7.69 ± 0.05	8.20 ± 0.46	7.55 ± 0.10
16:1^**Δ**9^	0.21 ± 0.04	0.17 ± 0.00	0.24 ± 0.01
18:0	3.46 ± 0.11	3.43 ± 0.16	3.45 ± 0.06
18:1^**Δ**9^	17.35 ± 0.07	*16.03 ± 0.34	16.91 ± 0.22
18:1^**Δ**11^	0.09 ± 0.03	0.07 ± 0.01	0.08 ± 0.02
18:2^**Δ**9^^**Δ**12^	27.79 ± 0.25	*27.29 ± 0.06	27.87 ± 0.12
18:3^**Δ**9^^**Δ**12^^**Δ**15^	16.04 ± 0.04	*17.70 ± 0.10	16.84 ± 0.05
20:0	2.01 ± 0.05	2.06 ± 0.03	2.18 ± 0.03
20:1^**Δ**11^	21.33 ± 0.18	21.00 ± 0.31	20.76 ± 0.13
20:1^**Δ**13^	1.70 ± 0.01	1.78 ± 0.02	1.79 ± 0.14
20:2^**Δ**9^^**Δ**12^	0.29 ± 0.02	0.33 ± 0.02	0.31 ± 0.01
22:0	0.23 ± 0.01	0.19 ± 0.01	0.18 ± 0.06
22:1^**Δ**13^	1.67 ± 0.05	1.65 ± 0.05	1.68 ± 0.02
24:0	0.14 ± 0.08	0.11 ± 0.05	0.15 ± 0.04
SFA^a^	13.53 ± 0.02	13.98 ± 0.57	13.52 ± 0.13
UFA^*b*^	86.47 ± 0.02	86.02 ± 0.57	86.48 ± 0.13
UFA/SFA	6.39 ± 0.01	6.16 ± 0.29	6.40 ± 0.07

*The data represent the mean ±SD of three independent replicates.*

*^a^Saturated fatty acid: 16:0 + 18:0 + 20:0 + 22:0 + 24:0.*

*^b^Unsaturated fatty acids and their derivatives: 16:1^Δ^ ^9^ + 18:1^Δ^ ^9^ + 18:1^Δ^ ^11^ + 18:2^Δ^ ^9Δ^ ^12^ + 18:3^Δ^ ^9Δ^ ^12Δ^ ^15^ + 20:1^Δ^ ^11^ + 20:1^Δ^ ^13^ + 20:2^Δ^ ^9Δ^ ^12^ + 22:1^Δ^ ^13^.*

*Significant differences (*p* < 0.05).

**FIGURE 9:**
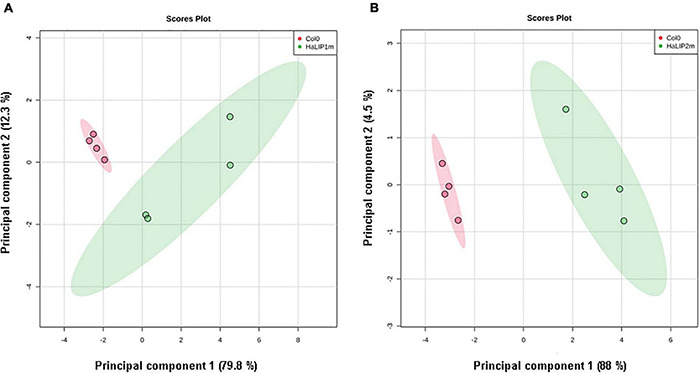
Principal component analysis (PCA) showing similar relationships between mature Arabidopsis WT Col-0 seeds and transgenic *HaLIP1m* **(A)** and *HaLIP2m* **(B)** seeds. The two principal components explain 92.1% (*HaLIP1m*) and 95.5% (*HaLIP2m*) of the variance. Controls, red circles and transgenic seeds, green circles.

**FIGURE 10:**
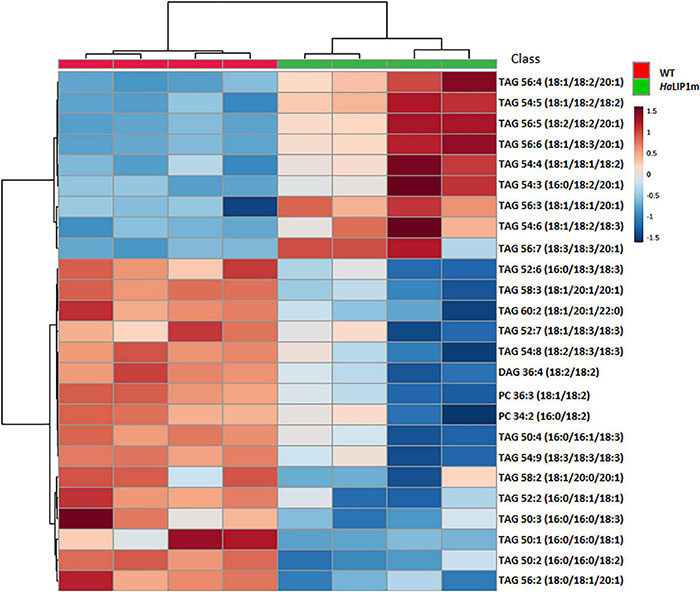
Hierarchical clustering and heatmap of the most significantly different lipid species (right) comparing mature control (WT, in red) and transgenic *HaLIP1m* seeds (blue). TOP 25 *p* < 0.05 *t*-test.

**FIGURE 11:**
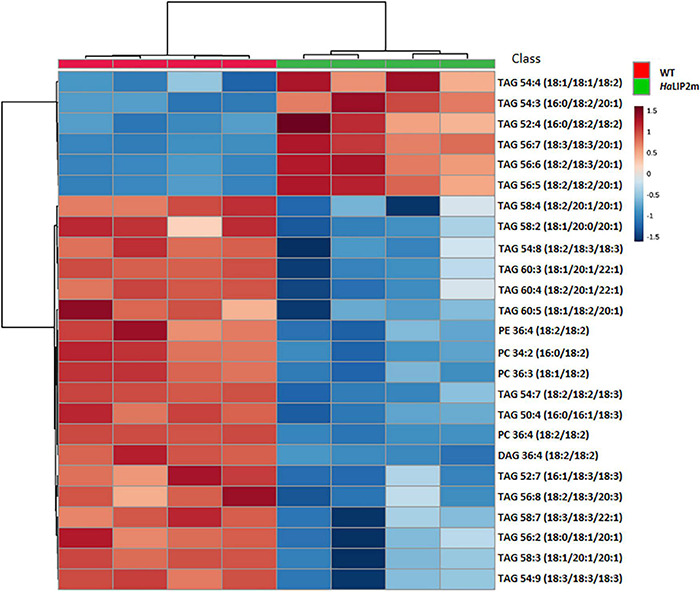
Hierarchical clustering and heatmap of the most significantly different lipid species (right) comparing mature control (WT, in red) and transgenic *HaLIP2m* seeds (blue). TOP 25 *p* < 0.05 *t*-test.

To gain further insights into the involvement of *Ha*LIP2m and *Ha*LIP1m in seed lipid biosynthesis, the lipidome of transgenic seeds overexpressing either *HaLIP1m* or *HaLIP2m* was analyzed. Despite the absence of an effect on the total fatty acid content in both transgenic lines, the content of some lipid species was altered by *HaLIP1m* or *HaLIP2m* overexpression, indicating a reorganization of the fatty acid distribution of the different lipid classes. In this study a total of 42 lipid species were identified and annotated, and an unsupervised PCA was initially employed to determine the experimental variation. In both cases, when the WT Col-0 lipidome was compared to the transformants, two separate groups of plots were detected that corresponded to the different genotypes (Figure 9). This overview of the lipid composition of the different seeds indicated that the different genotypes have distinct lipid compositions. A *t*-test was performed to detect the lipid species that were significantly different between the WT and each of the transgenic seed lines and hierarchical clustering was used to visualize these differences in heatmaps of the 25 seed lipids displaying the most significant differences (Figures 10, 11). A clear change in the content of some glycerolipid species was detected when both enzymes were overexpressed relative to the WT.

The overexpression of *HaLIP2m* affected some lipid species in seeds, such as TAGs species, producing an increase in TAGs 54:4, 54:3, 52:4, 56:7, 56:6, and 56:5, and a decrease in TAGs 58:4, 54:8, 54:7, 58:7, 56:2, 58:3, and PC 36:4 (Figure 11). Some of these TAG species represent a relevant component of the storage oil in seeds. The results indicated that overexpressing sunflower octanoyltransferase in Arabidopsis altered metabolism in a way that affected plant lipid biosynthesis. In this regard, an excess of octanoyltransferase in mitochondria could provoke a depletion of octanoyl-ACP within this organelle by interacting with this intermediate, competing with the endogenous mitochondrial KAS (Yasuno et al., 2004). This could restrict mitochondrial fatty acid synthesis, inducing a deficit of LA and lipid A. Defects in lipoylation have previously been reported in the *A. thaliana mtkas* mutant, deficient in mitochondrial KAS activity (Ewald et al., 2007). This mutation produced weaker H protein lipoylation from the glycine cleavage system, essential in photorespiration (Bauwe et al., 2010), since 95% of the complex is lipoylated through octanoyl-ACP produced by KAS activity via LIP2-LIP1 (Ewald et al., 2007). The restrictions in the lipoylated glycine system provoke metabolic alterations due to a decrease in the photorespiratory ability of the plant. These alterations affect the metabolism of cuticle formation and TAG synthesis, both at the level of gene expression and metabolism (Guan et al., 2017). Accordingly, the alterations caused by *HaLIP2m* overexpression could affect general lipid metabolism, which could in turn alter the distribution of fatty acid species in complex lipids, modifying the fatty acid metabolism of developing seeds. As a result, there may also be alterations in the pools of certain key metabolites like acyl-CoAs, which would modify the glycerolipid species accumulated based on their availability.

**FIGURE 12:**
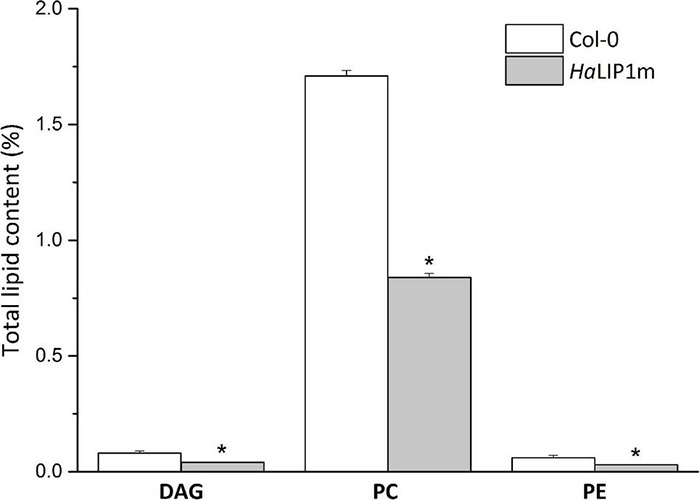
Relative (in reference to the total lipids) diacylglycerols (DAG), phosphatidyl choline (PC) and phosphatidylethanolamine (PE) in control *A. thaliana* seeds (white bars) and seeds transformed with *HaLIP1m* (gray bars). The data represents the average of three biological replicates and (*) indicates a significant difference (*p* < 0.05).

*HaLIP1m* overexpression also caused some variations in major TAG species in Arabidopsis seeds, provoking an increase in TAG 56:4, TAG 54:5, TAG 56:5, TAG 54:4, TAG 54:3, TAG 56:3, TAG 54:6, TAG 56:7, and a decrease in TAG 52:6, TAG 58:3, TAG 58:3, TAG 52:2, TAG 50:2, and TAG 56:2. In this case, the phosphatidylcholine (PC), phosphatidylethanolamine (PE) and diacylglycerol (DAG) glycerolipid content in transgenic *HaLIP1m* seeds was also altered, decreasing significantly in mature seeds (Figure 12). Thus, *HaLIP1m* overexpression decreased DAG to 50%, PC to 51%, and PE to 50%. These changes were analogous to those reported for the overexpression of the plastidial forms of this enzyme in Arabidopsis (*Ha*LIP1p1 and *Ha*LIP1p2: Martins-Noguerol et al., 2020), indicating that both enzymes had the same effect on plant lipid metabolism probably involved in the *de novo* biosynthesis of PC and PE. Thus, the glycerolipid biosynthesis of DAG, PC and PE is probably related, between them and with TAG synthesis (Li-Beisson et al., 2013). Thus, DAG is a precursor of PC and PE due to the action of amino alcohol phosphotransferases. These enzymes catalyze the biosynthesis of PC through CDP-choline by diacylglycerol cholinephosphotransferase and the biosynthesis of PE from CDP-ethanolamine by diacylglycerol ethanolaminephosphotransferase. Both CDP-derivatives are originated from PC or PE through the activity of choline-phosphate cytidyltransferase or CTP:phosphoethanolamine cytidyltransferase, respectively. PE can also be converted to PC by *N*-methyltransferase, which is dependent on SAM. Since *HaLIP1m* is also an enzyme that consumes SAM, its overexpression could cause the depletion of this co-factor in plant cells, restricting the PE/PC interconversion as previously hypothesized for sunflower plastidial LIP1 forms overexpressed in Arabidopsis seeds (Martins-Noguerol et al., 2020).

## Data Availability Statement

The original contributions presented in the study are included in the article/Supplementary Material, further inquiries can be directed to the corresponding author/s.

## Author Contributions

RM-N performed most of the experimental work. AM-P, SA, MT-P, and BT participated in the lipidomic analysis of mature seeds. RG, EM-F, JS, and AM-P participated in the work direction and experimental design. All the authors wrote and revised the manuscript.

## Conflict of Interest

The authors declare that the research was conducted in the absence of any commercial or financial relationships that could be construed as a potential conflict of interest.

## Publisher’s Note

All claims expressed in this article are solely those of the authors and do not necessarily represent those of their affiliated organizations, or those of the publisher, the editors and the reviewers. Any product that may be evaluated in this article, or claim that may be made by its manufacturer, is not guaranteed or endorsed by the publisher.

## Footnotes

1
https://www.heliagene.org/

2
http://swissmodel.expasy.org/

## Abbreviations

ACP: acyl carrier protein
BCDH: branched-chain 2-oxoacid dehydrogenase
DAF: days after flowering
DAG: diacylglycerol
FAMES: fatty acids methyl esters
FAS: fatty acid synthase
*HaLIP1m*: sunflower lipoyl synthase
*HaLIP2m*: sunflower octanoyltransferase
KAS: β-ketoacyl -(ACP) synthase
LA: lipoic acid
LIPA: bacterial lipoyl synthase
LIPB: bacterial octanoyltransferase
LIP1: plant lipoyl synthase
LIP2: plant octanoyltransferase
LPLA: lipoate protein ligase
2-OGDH: 2-oxoglutarate dehydrogenase
PC: phosphatidylcholine
PCA: principal component analysis
PDH: pyruvate dehydrogenase
PE: phosphatidylethanolamine
SAM: *S*-adenosylmethionine
TAG: triacylglycerols
WT: wild type.
