# Supplementary material for: Characterization of Helianthus annuus Lipoic Acid Biosynthesis: The Mitochondrial Octanoyltransferase and Lipoyl Synthase Enzyme System
Source: Front Plant Sci. 2021 Nov 18;12:781917. doi: 10.3389/fpls.2021.781917 (PMC8639206; doi:10.3389/fpls.2021.781917)
Supplement: Supplementary file 2 [file Data_Sheet_2.docx]

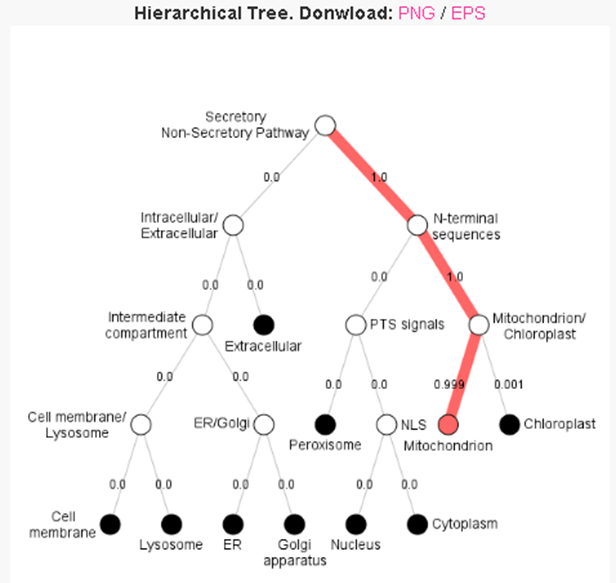


**Figure S1.** Hierarchical tree generated by the DeepLoc algorithm (Almagro-Armenteros et al., 2017) for *Ha*LIP1m protein location.

**Figure S2. (A)** Alignment of the sunflower octanoyltransferase (*Ha*LIP2m) amino acid sequence with homologous sequences from *Mycobacterium tuberculosis* (*Mt*LIPB; BAX49478.1) used for three-dimensional structure model. Identical evolutionarily conserved residues are highlighted in black and highly conserved residues are highlighted in grey. Residues involved in substrate binding are indicated with a green (Lys) or red arrow (Cys). The conserved PCG motif is indicated by a purple box. **(B)** Alignment of the sunflower lipoyl synthase (*Ha*LIP1m) amino acid sequence with homologous sequences from *Mycobacterium tuberculosis* (*Mt*LIPA; OHO19009.1) used for three-dimensional structure model. Strictly conserved residues are highlighted in black and highly conserved residues in grey. The [4Fe-4S] clusters that participate in the catalytic activity of LIP1 are marked by a red (RS cluster involved in SAM cleavage) and green box (auxiliary cluster with the Ser residue that also coordinates this cluster marked with a green arrow). The conserved R(S/T)S motif in the C-terminus is marked by a blue box.


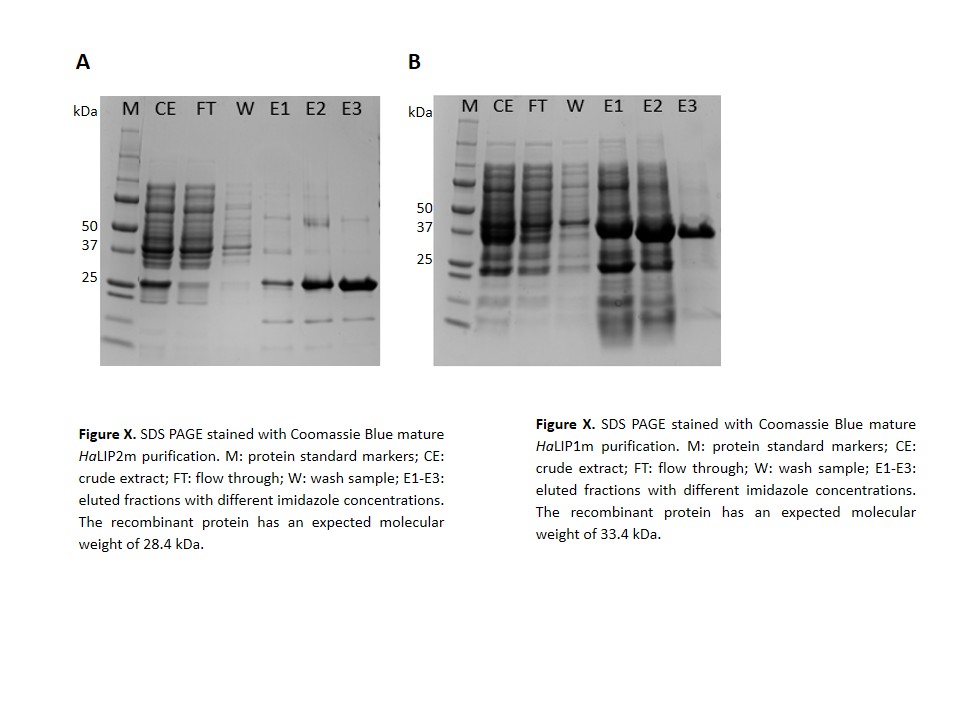


**Figure S3.** SDS PAGE of recombinant putative sunflower mitochondrial octanoyltransferase (*Ha*LIP2m) **(A)** and lipoyl synthase (*Ha*LIP1m) **(B)** stained with Coomassie Blue: M, protein standard markers; CE, crude extract; FT, flow through; W, wash sample; E1-E3, eluted fractions with different concentrations of imidazole (100, 200 and 500 mM). The mature recombinant proteins have an expected molecular weight of 28.4 kDa (*Ha*LIP2m) and 33.4 kDa (*Ha*LIP1m).


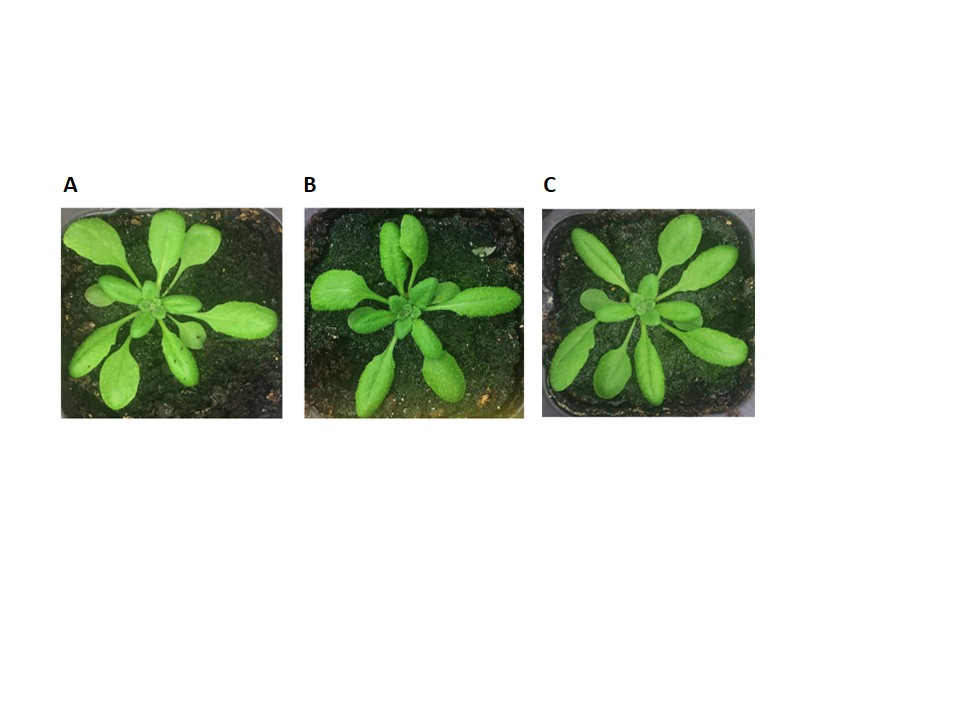


**Figure S4.** *A. thaliana* plants 26 days after germination. **(A)** Control Col-0. **(B)** Transgenic plants overexpressing putative sunflower mitochondrial lipoyl synthase (*Ha*LIP1m). **(C)** Transgenic plants overexpressing putative sunflower mitochondrial octanoyltransferase (*Ha*LIP2m).

**
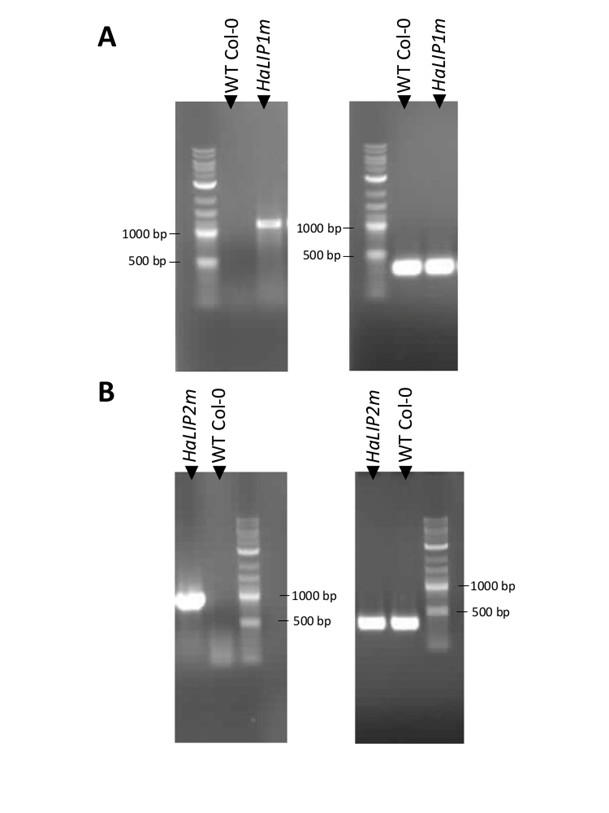
**

**Figure S5**. RT-PCR of transgenic *A. thaliana* plants overexpressing *HaLIP1m* **(A)** and *HaLIP2m* **(B)**. Panel left, RT-PCR shows presence of *HaLIP1m* **(A)** and *HaLIP2m* **(B)** transcripts in transgenic lines and absence of both transcripts in WT Col-0. Panel right, control signals from the constitutively expressed 18S ribosomal.

**Table S1.** **PCR primers used in this work.** *Ha*LIP1m, putative sunflower mitochondrial lipoyl synthase. *Ha*LIP2m, putative sunflower mitochondrial octanoyltransferase.

| **Primer** | **Sequence (5´- 3´) ^a^** |
| --- | --- |
| HaLIP1m-F-BamHI | TC**GGATCC**ATGCATTCTCGATTCAAATTTCTC |
| HaLIP1m-R-HindIII | GG**AAGCTT**TTACGCAGATGCAGCACG |
| HaLIP2m-F-BamHI | CCT**GGATCC**ATGAGAATTCCACGTAAACTTG |
| HaLIP2m-R-HindIII | GGC**AAGCTT**TCAACCATCAATTTCAAGATC |
| HaLIP1m-F-qpcr | CGCCACATGCCAGTTTCTG |
| HaLIP1m-R-qpcr | CCCGCTTTGTACGAAGACC |
| HaLIP2m-F-qpcr | GGTTTGGCGTATAACATTG |
| HaLIP2m-R-qpcr | CTTCATCAGGAAGCACC |
| HaActin-qpcr-F4 | GCTAACAGGAAAAGATGACT |
| HaActin-qpcr-R4 | ACTGGCATAAAGAGAAAGCACG |
| HaLIP2m-R-XbaI | GGTCTAGATCAACCATCAATTTCAAGATC |
| CAMV35S F1 | CGTAAGGGATGACGCACAAT |
| pBIN19-35S-R  At18S-F  At18S-R | CACACAGGAAACAGCTATGACC  GGTAGGCGATTGGCTAACATTGTCTGC  GAGACACCAACAGTCTTTCCTCTGCG |

**^a^** *Restriction sites are indicated in bold*

**Table S2.** Prediction of the subcellular location of the putative sunflower mitochondrial lipoyl synthase (*Ha*LIP1m) and octanoyltrasnsferase (*Ha*LIP2m). Data indicate the probability of a mitochondrial presequence.

N, non-mitochondrial

| **Algorithm** | ***Ha*LIP1m** | ***Ha*LIP2m** | ***At*LIP2m** | **Web Server References** |
| --- | --- | --- | --- | --- |
| Mitoprot | 0.97 | 0.44 | 0.13 | Claros and Vincens, 1996 |
| Predotar | 0.5 | 0.27 | N | Small et al., 2004 |
| PredSL | 0.99 | 0.44 | 0.86 | Petsalaki et al., 2006 |
| TPpred | Mito | N | N | Indio et al., 2013 |
| MitoFates | Mito | N | N | Fukasawa et al., 2015 |
| TargetP | 0.99 | 0.1 | 0.06 | Emanuelsson et al., 2007 |

**REFERENCES**

Claros, M. G, and Vincens, P. (1996). Computational method to predict mitochondrially imported proteins and their targeting sequences. *Eur J Biochem* 241, 779-786. doi: 10.1111/j.1432-1033.1996.00779.x

Small, I., Peeters, N., Legeai, F., and Lurin, C. (2004). Predotar: a tool for rapidly screening proteomes for N-terminal targeting sequences. *Proteomics* 4, 1581-1590.

Petsalaki, E. I., Bagos, P. G., Litou, Z. I., and Hamodrakas, S. J. (2006). PredSL: a tool for the N-terminal sequence-based prediction of protein subcellular localization. *Genomics Proteomics Bioinformatics* 4, 48-55. doi: 10.1016/S1672-0229(06)60016-8

Indio, V., Martelli, P. L., Savojardo, C., Fariselli, P., and Casadio, R. (2013). The prediction of organelle-targeting peptides in eukaryotic proteins with grammatical-restrained hidden conditional random fields. *Bioinformatics* 29, 981-988. doi: 10.1093/bioinformatics/btt089

Fukasawa, Y., Tsuji, J., Fu, S-C., Tomii, K., Horton, P., and Imai, K. (2015). MitoFates: improved prediction of mitochondrial targeting sequences and their cleavage sites. *Mol. Cell Proteomics* 14, 1113-1126. doi: 10.1074/mcp.M114.043083

Emanuelsson, O., Brunak, S., von Heijne, G., and Nielsen, H. (2007). Locating proteins in the cell using TargetP, SignalP and related tools. *Nat Protoc* 2, 953-971. doi: 10.1038/nprot.2007.131
